# A Mind-Body Physical Activity Program for Chronic Pain With or Without a Digital Monitoring Device: Proof-of-Concept Feasibility Randomized Controlled Trial

**DOI:** 10.2196/18703

**Published:** 2020-06-08

**Authors:** Jonathan Greenberg, Paula J Popok, Ann Lin, Ronald J Kulich, Peter James, Eric A Macklin, Rachel A Millstein, Robert R Edwards, Ana-Maria Vranceanu

**Affiliations:** 1 Integrated Brain Health Clinical and Research Program Massachusetts General Hospital Boston, MA United States; 2 Harvard Medical School Boston, MA United States; 3 Center for Pain Medicine Massachusetts General Hospital Boston, MA United States; 4 Department of Population Medicine Harvard TH Chan School of Public Health Boston, MA United States; 5 Biostatistics Center Massachusetts General Hospital Boston, MA United States; 6 Department of Psychiatry Massachusetts General Hospital Boston, MA United States; 7 Pain Management Center Brigham and Women's Hospital Boston, MA United States

**Keywords:** chronic pain, meditation, walking, feasibility studies, actigraphy

## Abstract

**Background:**

Chronic pain is associated with poor physical and emotional functioning. Nonpharmacological interventions can help, but improvements are small and not sustained. Previous clinical trials do not follow recommendations to comprehensively target objectively measured and performance-based physical function in addition to self-reported physical function.

**Objective:**

This study aimed to establish feasibility benchmarks and explore improvements in physical (self-reported, performance based, and objectively measured) and emotional function, pain outcomes, and coping through a pilot randomized controlled trial of a mind-body physical activity program (*GetActive*) with and without a digital monitoring device (*GetActive-Fitbit*), which were iteratively refined through mixed methods.

**Methods:**

Patients with chronic pain were randomized to the *GetActive* (n=41) or *GetActive-Fitbit* (n=41) programs, which combine relaxation, cognitive behavioral, and physical restoration skills and were delivered in person. They completed in-person assessments before and after the intervention. Performance-based function was assessed with the 6-min walk test, and step count was measured with an ActiGraph.

**Results:**

Feasibility benchmarks (eg, recruitment, acceptability, credibility, therapist adherence, adherence to practice at home, ActiGraph wear, and client satisfaction) were good to excellent and similar in both programs. Within each program, we observed improvement in the 6-min walk test (mean increase=+41 m, SD 41.15; *P*<.001; effect size of 0.99 SD units for the *GetActive* group and mean increase=+50 m, SD 58.63; *P*<.001; effect size of 0.85 SD units for the *GetActive-Fitbit* group) and self-reported physical function (*P*=.001; effect size of 0.62 SD units for the *GetActive* group and *P*=.02; effect size of 0.38 SD units for the GetActive-Fitbit group). The mean step count increased only among sedentary patients (mean increase=+874 steps for the *GetActive* group and +867 steps for the *GetActive-Fitbit* group). Emotional function, pain intensity, pain coping, and mindfulness also improved in both groups. Participants rated themselves as much improved at the end of the program, and those in the *GetActive-Fitbit* group noted that Fitbit greatly helped with increasing their activity.

**Conclusions:**

These preliminary findings support a fully powered efficacy trial of the two programs against an education control group. We present a model for successfully using the Initiative on the Methods, Measurement, and Pain Assessment in Clinical Trials criteria for a comprehensive assessment of physical function and following evidence-based models to maximize feasibility before formal efficacy testing.

**Trial Registration:**

ClinicalTrial.gov NCT03412916; https://clinicaltrials.gov/ct2/show/NCT03412916

## Introduction

### Background

Chronic musculoskeletal pain is costly [1] and associated with substantial emotional and functional limitations [2-5]. Current treatment recommendations support nonpharmacological approaches for pain management (Centers for Disease Control and Prevention guidelines) [6]. Evidence-based treatments such as cognitive behavioral therapy [7], acceptance and commitment therapy–based approaches [8], mindfulness-based approaches [9], and aerobic exercise [10] are efficacious in improving emotional and physical function. However, across all approaches, randomized controlled trials (RCTs) have tended to report mostly short-term improvements with relatively small effect sizes [9,11-16]. Novel interventions are needed to sustainably improve emotional and physical function in this population.

Using the International Classification of Functioning, Disability and Health (ICF) guidelines [17]; Initiative on Methods, Measurement, and Pain Assessment in Clinical Trials (IMMPACT) recommendations [18,19]; and guidelines for intervention development and clinical trials [20-22], we developed and iteratively refined a multimodal mind-body physical activity program that combines evidence-based pain management skills with increased physical activity through both quota-based pacing (gradual increase in activity that is noncontingent on pain levels) and linking increased walking to activities of daily living [23]. Our guiding hypothesis was that the most effective and efficient way to sustainably improve physical function among patients with chronic pain is to combine evidence-based mind-body skills with physical activity. In line with IMMPACT guidelines [18], the goal of the program is to increase not only self-reported but also performance-based (eg, the 6-min walk test [24]) and objectively measured (eg, step count as measured by accelerometers) physical function, which together provide a critical and comprehensive snapshot of an individual’s abilities and function. As previous research has shown that increasing activity in this population is challenging because of improper pacing [23,25], we hypothesized that Fitbit, an inexpensive, commercially available wrist-worn digital activity monitor with visible display, can be a useful aid by providing real-time self-monitoring and reinforcement of activity consistent with a predetermined weekly step goal. To date, several studies have used Fitbit alone, not paired with coping skills training, and did not find meaningful step count increases among patients with chronic pain [26,27]. We currently do not know whether daily monitoring of activity with a digital monitoring device such as Fitbit would aid participants in increasing step count.

Our long-term goal is to conduct a fully powered RCT comparing 2 identical mind-body physical activity programs, one with a Fitbit device (*GetActive-Fitbit*) and the other without (*GetActive*), with an attention educational placebo control. This future RCT will seek to (1) determine the efficacy of the 2 programs in sustainably improving self-reported, performance, and objectively measured physical function and to (2) understand whether program-dependent improvement in physical function (self-reported, performance based, and objectively measured) is enhanced by using a Fitbit. However, in accordance with the Obesity Related Behavioral Intervention Trials (ORBIT) [21] and the National Center for Complementary and Integrative Health (NCCIH) [22] models of intervention development and optimization, multiple program iterations are necessary to maximize methodology and feasibility before a large efficacy clinical trial. This study represents stage IIa and IIb of the ORBIT model (proof of concept and pilot testing) and stage 3 of the NCCIH treatment development model (feasibility and pilot studies). The goals of stages IIa and IIb of the ORBIT model are to test for clinically meaningful changes and determine the source of the treatment effect [21]. The goals of stage 3 of NCCIH’s development model are to determine whether a subsequent larger study of the refined intervention can be successfully implemented and can provide clinically meaningful evidence for efficacy [22].

We used a sequential approach to the development and initial testing of our mind-body physical activity program focused on multiple iterations to optimize it, meet a-priori set markers of feasibility, and establish a signal of improvement in outcomes (Multimedia Appendix 1) [21,28]. First, we conducted qualitative focus groups with adults with heterogeneous chronic pain to gather their feedback, needs and preferences, and barriers and facilitators to program participation, increasing activity and using a Fitbit [23]. Next, we developed a mind-body physical activity program that incorporates mind-body skills adapted from the Relaxation Response Resiliency Program [29], for example, eliciting the relaxation response (mindfulness meditation), pain-specific cognitive behavioral skills (eg, goal setting, behavioral activation techniques, adaptive restructuring of pain-related misconceptions such as catastrophizing and avoidance of fear), and physical restoration skills (eg, quota-based pacing noncontingent on pain). From this initial version, we developed the first version of *GetActive* (8 sessions), where participants increase activity using time-based goals, and *GetActive with Fitbit* (8 sessions), where participants increase activity using step count–based goals with real-time reinforcement aided by a Fitbit. Both programs focused on walking, as this was the preferred activity within our focus groups [23]. Using a nonrandomized, controlled, small pilot trial (ORBIT phase IIa; n=6 and n=7), we found that both programs had good to excellent feasibility and acceptability markers and relatively similar signals of improvement in physical, emotional function, and intervention targets [23]. However, exit interviews with group participants informed additional program modifications, including the use of self-compassion when goals are not met, language clarifications, and increasing the number of sessions to facilitate skill acquisition.

### Objectives

Here, we report on feasibility markers and within-group improvements in outcomes after a pilot RCT of the refined *GetActive* (10 group sessions) and *GetActive-Fitbit* (10 group sessions; Fitbit to self-monitor activity) programs. Our primary hypothesis was that both programs will meet a-priori set feasibility benchmarks (feasibility of recruitment, program acceptability, credibility and expectancy, therapist adherence to the manual, feasibility of quantitative measures, adherence to practice at home, adherence to ActiGraph accelerometer and Fitbit, and safety) necessary before an efficacy trial. Our secondary hypothesis was that for both programs, we will observe within-group improvements in physical function (self-reported, 6-min walk test, and ActiGraph step count), emotional function (anxiety and depression), pain-specific outcomes (intensity and coping with pain), as well as adaptive coping and mindfulness.

## Methods

### Participants

We recruited patients with heterogeneous musculoskeletal chronic pain via direct referrals from the Massachusetts General Hospital Pain Clinic as well as flyers and hospital-wide email lists advertising our study. Recruitment occurred between July 2018 and September 2019. The study was funded by the NCCIH and approved by Massachusetts General Hospital’s institutional review board (IRB). The inclusion criteria were as follows: (1) ≥18 years old, (2) self-reported nonmalignant chronic pain for >3 months, (3) ability to walk unassisted for at least 6 min, (4) access to a mobile device with Bluetooth version 4.0, (5) no change in psychotropic or pain medications for the past 3 months, and (6) cleared for participation by a physician. The exclusion criteria were as follows: (1) medical illness expected to worsen in the next 6 months, (2) serious and untreated psychiatric illness or active suicidality, (3) current untreated substance use disorder, (4) practicing meditation/yoga or relaxation response skills for >45 min a week in the past 6 months, (5) using a Fitbit device in the past 6 months, and (6) engaged in regular physical exercise for >30 min daily by self-report.

### Procedure

#### Screening, Enrollment, and Randomization

We screened all referrals via a phone call using a standard, IRB-approved scripted checklist. All participants were informed that the goal of the program was to increase physical activity rather than decrease pain. We documented all reasons for ineligibility (Multimedia Appendix 2). All screenings were reviewed at team meetings with the study principal investigator. Eligible participants who were able to meet during the designated group times (a 3-hour block that would accommodate randomization to *GetActive* or *GetActive-Fitbit* 90-min groups) were immediately scheduled for the next available group sessions. The rest were placed on a waitlist and contacted for future groups. The date and time for each group cohort was flexibly determined based on the availability of most eligible potential participants. For each group cohort, participants were asked to come to the clinic on the same day and time to undergo informed consent, complete self-report assessments, complete a 6-min walk test [24], and start wearing a wGT3X-BT ActiGraph accelerometer (ActiGraph, LLC). Participants received detailed instructions (1) to wear the ActiGraph over their right hip using an elastic belt for 1 week during all waking hours, except while in water (bathing or swimming), (2) to maintain their regular levels of activity, and (3) to fill out a daily device wear and physical activity log. Participants were asked their preferred method to receive daily reminders to wear the ActiGraphs (eg, text messages, phone calls, or emails). At the end of the assessment session (approximately 90 min), participants were randomized to either the *GetActive* or *GetActive-Fitbit* program based on a 1:1 ratio via a sequence generated by *sealedenvelope.com* in blocks of 12. Participants were compensated with US $30 for completion of baseline assessments. They were instructed to return for the first session the following week at their assigned time and return the ActiGraph and wear log. In the first session, participants received the *GetActive* or *GetActive-Fitbit* treatment manuals. Those randomized to *GetActive-Fitbit* also received a Fitbit that was paired with each participant’s phone. The average step count recorded by the ActiGraph was set as the initial step goal on the participants’ Fitbit devices.

#### GetActive and GetActive-Fitbit Programs

Development of the original 8-week programs, details on program skills, and exit interviews to inform the current program versions have been previously reported [23]. The final *GetActive* and *GetActive-Fitbit* programs have 10 weekly 90-min sessions (Multimedia Appendix 3). The programs teach 4 core skills: (1) weekly SMART goal setting (defined as goals that are specific, measurable, achievable, relevant and time-based) [29] for a gradual increase in physical activity paired with activities of daily living that are meaningful and important to participants (ie, walk instead of drive to the store and walk to the park with kids) and the daily practice of mind-body skills (eg, engage in meditation before going to bed and when walking), (2) individualized quota-based pacing (eg, walk for 30 min or meet a step goal of 5000), (3) mind-body skills (diaphragmatic breathing to manage intense pain flares and pain anxiety, body scan to increase body awareness and reduce reactivity to pain sensations, mindfulness exercises to understand the transience of pain and change one’s relationship with it, and self-compassion when falling short of set goals), and (4) understand the disability spiral (eg, how reducing activity perpetuates pain and disability) and correct myths about pain or automatic pain-related thoughts that interfere with meeting program goals. At each weekly session, the group leader reviewed home practice, including adherence to activity goals, and helped participants solve barriers to adherence. Participants who missed group sessions were immediately contacted by the study staff and scheduled for a make-up session.

The *GetActive* and *GetActive-Fitbit* programs are identical in content and structure. However, in the *GetActive-Fitbit* program, the study staff instructed participants how to consistently wear and charge the Fitbit (session 1), uploaded an individualized step goal onto each participant’s Fitbit during each weekly session through Fitbit.com, monitored Fitbit wear in real time through the Fitabase website (Fitabase) [30], immediately called nonadherent participants to solve problems with adherence, and encouraged participants to focus on meeting the daily activity SMART goals.

#### Procedure for Fitbit Step Count Assessments and Pacing

Details can be found in the study by Greenberg et al [23]. Briefly, before each session, a staff member downloaded the participants’ Fitbit data from the past week, calculated the participants’ step goals for the upcoming week, and provided the study clinician with individual adherence data for each participant, which were discussed during the session. If participants reached their weekly goal, they could choose whether to repeat it for the following week or increase it by 10% [31,32]. If they did not reach their goal, they would repeat it. If they did not reach their goal 2 weeks in a row, they could choose whether to repeat it or set a new goal that was 10% higher than their actual step count during that week (rather than their step count goal).

#### Postintervention Assessments

In the last group session, participants were handed the ActiGraph and were instructed to complete the activity log again for 1 week. They were asked to return the following week as a group to complete the postintervention assessment. Participants were again compensated with US $30 for the assessment session.

#### Feasibility Assessments

Feasibility markers were determined to be consistent with guidelines for intervention development [21,28]. Feasibility benchmarks were set a-priori. We assessed the following feasibility markers:

1. *Feasibility of recruitment:* This was assessed as the proportion of potential participants successfully contacted who agreed to participate. We considered a proportion of 80% excellent and a proportion of 70% good.
2. *Program acceptability:* This was assessed via the proportion of participants who attended at least 7 out of 10 sessions. Feasibility was considered excellent when >80% of the participants attended 7 out of 10 sessions and good if at least 70% of the participants did.
3. *Credibility and expectancy:* This was assessed using the credibility and expectancy questionnaire [33], a 6-item questionnaire assessing both the degree to which participants find the program logical and convincing (credibility) and the degree to which they believe they will benefit (expectancy). Credibility and expectancy were considered excellent when >75% of the participants scored above the scale midpoint and good if at least 70% of the participants did.
4. *Therapist adherence to the program manual:* This was assessed in 2 ways. First, the study clinician completed a content checklist at the end of each of the sessions, marked all session content that was covered within each session, and wrote a progress note. Therapist adherence to the program manual was determined as excellent if 100% of the therapist checklists covered 100% of the session skills and good if at least 75% of the checklists covered 100% of the session skills. Next, 20% of the audio recorded sessions were coded for content against the therapist checklists by 2 independent coders who were trained by the principal investigator. An agreement (Cohen κ) of at least 60% was considered good and higher than 80% was considered excellent [34].
5. *Feasibility of quantitative measures:* This was measured by assessing the questionnaire completion and internal consistency reliability of the measures. This was considered to be acceptable if the internal reliability of the questionnaires (Cronbach α) was higher than .70 and none of the questionnaires were fully missing in more than 25% of the participants.
6. *Adherence to home practice:* Adherence to home practice was assessed via the number of days per week participants self-reported that they practiced meditation and appreciation exercises. We considered adherence to be excellent if 3 home practice components (eg, relaxation response practice, SMART goal activity, and appreciations) were completed at least 3 out of 7 days per week or if they included at least one of the 3 components at least 5 out of 7 per week on average, in 80% of home practice logs handed in. We considered adherence to activity and home practice to be good if these criteria were met in 70% of logs.
7. *Adherence to ActiGraph and Fitbit:* This was calculated by the proportion of participants who wore the ActiGraph at least 5 out of 7 days per week [35,36] for at least seven hours a day (≥80% excellent and ≥70% good) [23]. Similar criteria were used for wearing the Fitbit in the *GetActive-Fitbit* group.
8. *Adherence to Fitbit:* For the *GetActive-Fitbit* group, adherence to Fitbit was calculated by the proportion of participants who wore a charged Fitbit for 5 out of 7 days per week on average (≥80% excellent and ≥70% good).
9. *Program satisfaction:* This was assessed using the client satisfaction scale questionnaire (CSQ-3) [37], which includes 3 items capturing the degree to which the program met the participants’ needs and their satisfaction from it. Satisfaction was considered excellent if the proportion of participants who scored above the scale midpoint was ≥75% and good if ≥70%.
10. *Program safety:* Safety was assessed via self-reported adverse events. Safety was considered excellent if there were no adverse events linked to program participation and good if there were minimal and mild adverse events (eg, muscle soreness) linked to program participation, which occurred in no more than 10% of participants.

### Physical Function Assessments

We assessed objectively measured, performance-based, and self-reported physical function per the IMMPACT criteria [18,19] and the ICF guidelines [17].

#### Objectively Measured Physical Function

We measured the actual number of steps taken daily by each participant using the *wGT3X-BT ActiGraph accelerometer* device [38]. We asked participants to wear the ActiGraph during all waking hours for 7 days before and after the programs and calculated the participants’ average daily steps. We used ActiLife software (ActiLife LLC) to store, clean, and analyze data using the settings used in our nonrandomized controlled trial [23]. Briefly, the ActiGraphs were set to record counts in 30-second epochs [39]. Nonwear time was defined as ≥90 consecutive minutes of 0 activity counts [40]. Up to 2 min of activity counts between 0 and 100 were allowed [41], and we ignored wear periods of <10 min. A staff member checked for valid wear time (7 hours per day) and manually checked each participant’s recorded activity with self-reported wear times logged in their activity diaries to ensure consistency. We used a minimum clinically important difference (MCID) [42] of 800 ActiGraph measured steps [43].

#### Performance-Based Physical Function

We measured performance-based physical function via the 6-min walk test [44], which has an MCID of 54 m [44]. We recorded the distance in meters each participant covered by walking on a flat surface for 6 min.

#### Self-Reported Physical Function

We used 3 self-report measures recommended by IMMPACT [18] that assess different aspects of physical function targeted within our program. The *World Health Organization Disability Assessment Schedule (WHODAS) 2.0* is a 36-item questionnaire assessing difficulties in 6 main areas of function: cognition (understanding and communication), mobility (moving and getting around), self-care (hygiene, dressing, eating, and staying alone), getting along with others (interacting with other people), life activities (domestic responsibilities, leisure, and work), and participation (joining community activities) [45]. WHODAS 2.0 does not yet have an established MCID. The *Patient-Reported Outcomes Measurement Information System (PROMIS) physical function*, version 1.2.8b, is an 8-item questionnaire that assesses the ability to perform various physical tasks (self-care to complex tasks) [46]. Scores are expressed as T scores (mean 50, SD 10). The MCID is 5.48 [47]. The self-reported *physical activity scale for individuals with physical disabilities (PASIPD)* is a 13-item measure assessing engagement in leisure, household, and work-related physical activities [48]. Internal reliability in the current sample was excellent for the PROMIS physical function and WHODAS (Cronbach α=.94 and .97, respectively). For the PASIPD, reliability was acceptable (α=.62).

#### Emotional Function

We measured anxiety using the PROMIS anxiety scale [49] (*version 1.08a*, MCID=4.28) and depression using the PROMIS depression scale [50] (*version 1.08b*, MCID=5.19) [47,49,50]. These 8-item measures assess the frequency of anxiety and depression symptoms, respectively, over the past week on a 1-5 Likert scale. Scores are expressed as T scores with mean 50 (SD 10). Internal reliability was excellent for PROMIS anxiety (Cronbach α=.95) and depression (Cronbach α=.96).

#### Pain Intensity

We assessed pain intensity at rest and with activity using the *numerical rating scale* (NRS) [51,52]. This is a 0-10 scale with high scores depicting higher pain. The NRS has an MCID of 1 [53].

#### Pain-Related Coping

We assessed pain-related coping using the *pain catastrophizing scale* [54], which assesses hopelessness, helplessness, and magnification of pain; the *Tampa kinesiophobia scale* (MCID=6) [55], which assesses fear of experiencing pain during activity; and the *pain resilience scale* (no MCID) [56], which measures the ability to regulate emotions and engage in activities despite pain. Internal reliability was excellent for pain catastrophizing (Cronbach α=.94) and good for both kinesiophobia (Cronbach α=.87) and pain resilience (Cronbach α=.89).

#### Adaptive Coping and Mindfulness

We assessed adaptive coping skills using the *measure of current status* (MOCS-A) [57], a 13-item questionnaire measuring the ability to utilize healthy coping skills such as relaxation, awareness of tension, assertiveness, and coping confidence. We assessed mindfulness using the *cognitive and affective mindfulness scale-revised* (CAMS-R), a 12-item questionnaire measuring participants’ ability to pay attention to the present moment in a nonjudgmental manner [58]. Internal reliability was good for both measures (Cronbach α=.89 for MOCS-A and Cronbach α=.85 for CAMS-R).

#### Patients’ Perception of Improvement

We used the *modified patient global impression of change* (MPGIC) to assess participants’ overall perception of improvement in key program areas [59]. Participants used a 1-7 Likert scale to rate how much they perceived the program to have improved their physical function, activity levels, pain, emotional function, pain resilience, and the degree to which the Fitbit helped them or not in increasing physical activity (only the *GetActive-Fitbit* group). Lower scores indicate *higher* perceived improvement.

#### Analysis Plan

We first assessed the sample characteristics using descriptive statistics. We then analyzed feasibility markers based on the proportion of participants who achieved each benchmark, as detailed earlier in the *Feasibility Assessments* section. For the rest of the quantitative assessments, we used descriptive statistics to characterize the sample and paired sample two-tailed *t* tests to assess within-group changes between baseline and postprogram. Cohen d was used to determine effect sizes using conventional standards (small effect sizes of 0.2 SD units, medium effect sizes of 0.5 SD units, and large effect sizes of 0.8 SD units) [60]. When available, we report clinical significance based on MCID and refer to comparisons with population norms. Consistent with recommendations for analyses for pilot studies [61,62], we did not perform between-group analyses of efficacy.

#### Sample Size Consideration

In line with recommendations for pilot RCTs [63,64], the target sample size for this study was 80 enrolled participants to achieve 60 completers. This sample size is sufficient to determine feasibility and acceptability markers and is typical in randomized controlled feasibility trials [65-67].

## Results

### Sample Characteristics

A total of 265 participants were referred and assessed for eligibility, and 82 participants were randomized (41 in each group; Multimedia Appendix 2). The sample characteristics are summarized in Table 1. Participants were predominantly female (54/82, 66%), white (66/82, 80%), and non-Hispanic (72/82, 88%). Approximately half of all participants completed either 4 years of college (17/82, 21%) or obtained a graduate or professional degree (28/82, 34%).

**Table 1:** Demographic characteristics of participants.

Demographic characteristics			GetActive (n=41)		GetActive-Fitbit (n=41)
Age (years), mean (SD)			54.46 (14.5)		49.07 (14.2)
**Gender, n (%)**					
	Male	18 (43.9)		10 (24.4)	
	Female	23 (56.1)		31 (75.6)	
**Ethnicity, n (%)**					
	Hispanic or Latino/Latina	4 (9.8)		4 (9.8)	
	Not Hispanic or Latino/Latina	35 (85.4)		37 (90.2)	
	Missing	2 (4.9)		N/A^a^	
**Race, n (%)**					
	American Indian/Alaskan Native	2 (4.9)		0 (0.0)	
	Asian	1 (2.4)		2 (4.9)	
	Black/African American	5 (12.2)		2 (4.9)	
	Native Hawaiian/Pacific Islander	0 (0.0)		0 (0.0)	
	White	32 (78.0)		34 (82.9)	
	More than one race	1 (2.4)		3 (7.3)	
**Marital status, n (%)**					
	Single, never married	13 (31.7)		15 (36.6)	
	Living with significant other	5 (12.2)		6 (14.6)	
	Married	15 (36.6)		8 (19.5)	
	Separated/divorced	5 (12.2)		11 (26.8)	
	Widowed	3 (7.3)		1 (2.4)	
**Education, n (%)**					
	High school (12 years)	5 (12.2)		6 (14.6)	
	Some college/associate degree (<16 years)	15 (36.6)		11 (26.8)	
	Completed college (16 years)	9 (22.0)		8 (19.5)	
	Graduate/professional degree (>16 years)	12 (29.3)		16 (39.0)	

^a^N/A: data not applicable.

### Feasibility and Acceptability Markers

Program acceptability, feasibility of quantitative measures, therapist adherence, patient adherence to activity and home practice, adherence to wearing the ActiGraph (as well as Fitbit for the *GetActive-Fitbit* program), and program satisfaction were good to excellent and similar in both groups (Table 2). Feasibility of recruitment was excellent, with 265 participants out of 307 (86.3%) successfully contacted agreeing to participate). 9 out of 10 multi-item measures had internal reliability (Cronbach α) >.70, with the mean reliability=0.89, SD 0.11. Of the 82 participants randomized, baseline ActiGraph data were obtained from 72 participants (68 with valid data: 31 in the *GetActive* program and 37 in the *GetActive-Fitbit* program) and postprogram data from 60 participants (56 with valid data: 25 in the *GetActive* program and 31 in the *GetActive-Fitbit* program). The 6-min walk test data were obtained from 82 participants at baseline (41 in each group) and 61 postprogram (28 in the *GetActive* program and 33 in the *GetActive-Fitbit* program). Complete data from all self-reported measures were obtained from 81 participants at baseline (41 from the *GetActive* program and 40 in the *GetActive-Fitbit* program) and from 70 (34 from the *GetActive* program and 36 in the *GetActive-Fitbit* program) participants postprogram. The benchmark criteria are detailed in the *Feasibility Assessments* section.

**Table 2:** Feasibility and acceptability of the programs.

Outcomes^a^	GetActive	GetActive-Fitbit
Program acceptability	31 out of 41 participants (76%) attended ≥7 out of 10 group or make-up sessions: good	34 out of 41 participants (83%) attended ≥7 out of 10 group or make-up sessions: excellent
Credibility and expectancy	27 out of 41 participants (66%) scored above the scale midpoint for expectancy: acceptable; 37 out of 41 participants (90%) scored above the scale midpoint for credibility: excellent	38 out of 41 participants (93%) scored above the scale midpoint for credibility: excellent; 22 out of 41 participants (54%) scored above the scale midpoint for expectancy: acceptable
Therapist adherence to the manual	Rater agreement (κ)=98%, therapist adherence to the manual was 98%: good	Rater agreement (κ)=97%, therapist adherence to the manual was 94%: good
Feasibility of quantitative measures	41 out of 41 (100%) were not fully missing questionnaires on quantitative measures at baseline: excellent; 34 out of 35 (97%) were not fully missing questionnaires on quantitative measures at posttest: excellent	40 out of 41 (97.56%) were not fully missing questionnaires on quantitative measures at baseline: excellent; 37 out of 37 (100%) were not fully missing questionnaires on quantitative measures at posttest: excellent
Adherence to homework	98% of logs handed in met adherence criteria (ie, 3 home practice components completed 3 out of 7 days per week or 1 component completed 5 out of 7 days per week): excellent	98% of logs handed in met adherence criteria (ie, 3 home practice components completed 3 out of 7 days per week or 1 component completed 5 out of 7 days per week): excellent
Adherence to ActiGraphs and Fitbit	31 out of 33 participants (94%) who received the ActiGraph at baseline wore it for ≥5 out of 7 days: excellent; 25 out of 27 participants (93%) who received the ActiGraph at posttest wore it for 5 out of 7 days: excellent; 30 out of 33 (91%) participants who received the ActiGraph at baseline had at least 5 out of 7 valid days (minimum of 7 wear hours): excellent; 19 out of 27 (70%) participants who received the ActiGraph at posttest had at least 5 out of 7 days (minimum of 7 wear hours): good	36 out of 39 participants (92%) who received the ActiGraph at baseline wore it for ≥5 out of 7 days: excellent; 29 out of 33 participants (88%) who received the ActiGraph at posttest wore it for 5 out of 7 days: excellent; 28 out of 39 participants (72%) who received the ActiGraph at baseline had at least 5 out of 7 valid (minimum of 7 wear hours): good; 25 out of 33 participants (76%) who received the ActiGraph posttest had at least 5 out of 7 valid (minimum of 7 wear hours): good; 34 out of 41 participants (83%) wore the Fitbit for at least 5 out of 7 days for 8 out of 10 weeks of the program: good
Client satisfaction	34 out of 34 participants (100%) scored above the scale midpoint: excellent	35 out of 36 participants (97%) scored above the scale midpoint: excellent
Program safety and adverse events	3 participants were hospitalized for reasons unrelated to the program (1 for a lung infection, 1 for a pain flare, and 1 for a stroke); 1 patient reported Sciatica: excellent	7 participants were hospitalized for reasons unrelated to the program (1 for sickle cell anemia flare-up; 1 for diverticulitis attack; 1 for chemotherapy; 1 for a pain flare; 1 for unknown reasons; 1 for falling; and 1 admitted twice for elevated heartrate, and then hurting leg); 1 patient reported a sprained quadricep muscle: good

^a^Feasibility of recruitment and internal-reliability portion of the feasibility of quantitative measures are reported in the text for the entire sample.

### Physical Function

#### Objective Measured Physical Function

As a whole, neither the *GetActive* group (–191 steps; *P=*.68; effect size of 0.09 SD units) nor the *GetActive-Fitbit* group (+8 steps; *P=*.98; effect size of 0 SD units) exhibited increases in step count from pre- to postprogram (Table 3). A total of 48% (11/23) of participants in the *GetActive* group and 53% (16/30) of participants in the *GetActive-Fitbit* group exhibited a higher step count following the program compared with baseline, with mean improvements exceeding the MCID of 800 steps (+1341 steps for the *GetActive* group and +1441 for the *GetActive-Fitbit* group). There was wide variability in the baseline step count across participants (mean=5432 SD~2942 steps; range 1197-13,643 steps). Although our goal was to enroll individuals who were inactive, 47% of individuals in our sample recorded >5000 steps at baseline (48% in the *GetActive* group and 46% in the *GetActive-Fitbit* group). Within the subgroup of participants who were sedentary (ie, recorded <5000 steps at baseline), the mean change in number of steps was above the MCID in both the *GetActive* and *GetActive-Fitbit* groups (+874 steps; *P=*.25; effect size of 0.39 SD units for the *GetActive* group and +867 steps; *P=*.03; effect size of 0.56 SD units for the *GetActive-Fitbit* group). In all, 50% of the sedentary individuals in the *GetActive* group and 47% in the *GetActive-Fitbit* group increased the number of steps over MCID.

#### Performance-Based Physical Function

Participants in both the *GetActive* group (+41 m; *P<*.001; effect size of 0.99 SD units) and *GetActive-Fitbit* group (+50 m; *P<*.001; effect size of 0.85 SD units) improved significantly with large effect sizes on the 6-min walk test, and these were just at the cusp of the MCID for the *GetActive-Fitbit* group.

#### Self-Reported Physical Function

Both groups exhibited significant improvements with medium effect size in both the WHODAS (*P=*.001; effect size of 0.62 SD units for the *GetActive* group and *P=*.03; effect size of 0.38 SD units for the *GetActive-Fitbit* group) and PROMIS physical function (*P=*.01; effect size of 0.49 SD units for the *GetActive* group and *P=*.02; effect size of 0.40 SD units for the *GetActive-Fitbit* group). Differences in the PROMIS measure (for which the MCID is available) did not reach the MCID. Groups exhibited no change in self-reported physical activity measured by the PASIPD (*P=*.32; effect size of 0.17 SD units for the *GetActive* group and *P=*.56; effect size of 0.09 SD units for the *GetActive-Fitbit* group).

### Emotional Function

The *GetActive* group exhibited nonsignificant improvements in small effect size for the *PROMIS depression* (*P=*.11; effect size of 0.27 SD units) and *PROMIS anxiety* (*P=*.08; effect size of 0.30 SD units). The *GetActive-Fitbit* group exhibited significant improvements in medium effect sizes in the *PROMIS depression* (*P=*.003; effect size of 0.54 SD units) and *PROMIS anxiety* (*P=*.02; effect size of 0.40 SD units).

### Pain Intensity

Both groups exhibited clinically meaningful and significant reductions in medium effect size in pain at rest (*P=*.007; effect size of 0.49 SD units for the *GetActive* group and *P=*.001; effect size of 0.58 SD units for the *GetActive-Fitbit* group) and with activity (*P<*.001; effect size of 0.70 SD units for the *GetActive* group and *P=*.001; effect size of 0.59 SD units for the *GetActive-Fitbit* group).

### Pain-Related Coping

Both groups showed significant and medium-sized effects for improvements in pain catastrophizing (*P<*.001; effect size of 0.72 SD units for the *GetActive* group and *P=*.01; effect size of 0.43 SD units for the *GetActive-Fitbit* group) and kinesiophobia (*P<*.001; effect size of 0.74 SD units for the *GetActive* group and *P=*.001; effect size of 0.61 SD units for the *GetActive-Fitbit* group). The *GetActive* group exhibited significant medium-sized improvements in pain resilience (*P=*.001; effect size of 0.64 SD units), whereas improvements in the *GetActive-Fitbit* group had a small effect size and did not reach significance (*P=*.09; effect size of 0.28 SD units).

### Adaptive Coping and Mindfulness

Both groups exhibited significant medium-sized effects for improvements in mindfulness (*P=*.002; effect size of 0.58 SD units for the *GetActive* group and *P=*.01; effect size of 0.44 SD units for the *GetActive-Fitbit* group) and significant medium-to-large effects for improvement in adaptive coping (*P<*.001; effect size of 0.76 SD units for the *GetActive* group and *P<*.001; effect size of 0.83 SD units for the *GetActive-Fitbit* group).

### Patients’ Perception of Improvement

Perceived improvement in pain, physical activity, and physical and emotional function were high for both programs. Improvement due to Fitbit was high in the *GetActive-Fitbit* group (Table 4).

**Table 3:** Outcome measures.

Measures	GetActive						GetActive-Fitbit					
	Baseline, mean (SD)	Posttest, mean (SD)	Pretest-posttest change, mean (95% CI)	*t* test value (df)	*P* value	Cohen *d*	Baseline, mean (SD)	Posttest, mean (SD)	Pretest-posttest change, mean (95% CI)	*t* test value (df)	*P* value	Cohen *d*
ActiGraph average steps	6330.57 (3556.10)	6139.25 (3424.39)	191.32 (−758.56 to 1141.19)	0.42 (22)	.68	0.088	5447.55 (2597.42)	5455.48 (2320.37)	−7.93 (−705.02 to 689.16)	−0.023 (29)	.98	−0.00
6-min walk test distance (m)	358.393 (85.11)	399.14 (77.19)	−40.75 (−56.71 to −24.79)	−5.24 (27)	<.001	−0.99	343.83 (77.56)	393.58 (71.88)	−49.74 (−70.53 to −28.95)	−4.87 (32)	<.001	−0.85
Physical function (PROMIS^a^)	40.55 (6.63)	42.97 (7.46)	−2.41 (−4.10 to −0.72)	−2.90 (34)	.006	−0.49	38.10 (7.86)	40.76 (8.41)	−2.65 (−4.86 to −0.44)	−2.44 (36)	.02	−0.40
Physical function (WHODAS^b^)	25.02 (15.52)	17.45 (13.72)	7.56 (3.39 to 11.73)	3.684 (34)	.001	0.62	34.65 (21.02)	27.13 (16.70)	7.52 (0.96 to 14.09)	2.32 (36)	.03	0.38
Self-reported physical activity	9.60 (7.04)	11.15 (8.51)	−1.55 (−4.64 to 1.55)	−1.02 (34)	.32	−0.17	15.37 (18.65)	17.13 (14.93)	−1.76 (−7.89 to 4.37)	−0.583 (36)	.56	−0.09
Depression	51.63 (10.34)	49.34 (9.29)	2.29 (−0.58 to 5.16)	1.62 (34)	.11	0.27	59.47 (9.53)	55.60 (7.72)	3.87 (1.45 to 6.28)	3.25 (35)	.003	0.54
Anxiety	52.48 (10.14)	50.81 (8.52)	2.87 (−0.40 to 6.14)	1.78 (34)	.08	0.30	58.87 (9.07)	56.12 (9.50)	2.95 (0.49 to 5.42)	2.43 (36)	.02	0.40
Pain at rest	4.77 (2.51)	3.57 (2.5)	1.2 (0.35 to 2.04)	2.89 (34)	.007	0.49	5.78 (2.18)	4.56 (2.16)	1.22 (0.51 to 1.94)	3.47 (35)	.001	0.58
Pain with activity	6.63 (1.99)	4.97 (2.54)	1.66 (0.85 to 2.46)	4.17 (34)	<.001	0.70	7.24 (2.23)	6.08 (2.55)	1.16 (0.51 to 1.81)	3.62 (36)	.001	0.59
Pain resilience	37.57 (9.65)	43.49 (9.99)	−5.91 (−9.10 to −2.73)	−3.77 (34)	.001	−0.64	33.16 (10.07)	36.46 (11.11)	−3.30 (−7.24 to 0.64)	−1.70 (36)	.09	−0.28
Pain catastrophizing	18.34 (11.06)	9.77 (7.66)	8.57 (4.47 to 12.67)	4.24 (34)	<.001	0.72	21.41 (11.94)	16.36 (10.84)	5.03 (1.13 to 8.93)	2.61 (36)	.01	0.43
Kinesiophobia	37.23 (7.72)	30.76 (7.36)	6.46 (3.41 to 9.52)	4.30 (33)	<.001	0.74	38.86 (8.87)	34.78 (8.25)	4.08 (1.84 to 6.32)	3.70 (36)	.001	0.61
Adaptive coping	28.49 (1.56)	36.37 (1.44)	−7.89 (−11.43 to −4.34)	−4.52 (34)	<.001	0.76	27.08 (1.65)	34 (1.38)	−6.92 (−9.69 to −4.15)	−5.07 (36)	<.001	0.83
Mindfulness	32.57 (6.58)	36.31 (6.48)	−3.74 (−5.96 to −1.53)	−3.43 (34)	.002	−0.58	30.43 (7.02)	33.14 (6.18)	−2.70 (−4.76 to −0.64)	−2.66 (36)	.012	−0.44

^a^PROMIS: Patient-Reported Outcomes Measurement Information System.

^b^WHODAS: World Health Organization Disability Assessment Schedule.

**Table 4:** Perceived improvement of change (1=very much improved and 7=very much worse).

Group	Impression of change in pain	Impression of change in physical activity	Impression of change in physical function	Impression of change in emotional function	Impression of change in resiliency	Impression of change from use of Fitbit
GetActive, mean (SD)	2.51 (1.17)	2.31 (1.18)	2.57 (1.19)	2.40 (1.01)	2.14 (0.91)	N/A^a^
GetActive-Fitbit, mean (SD)	2.70 (1.20)	2.30 (1.10)	2.78 (0.95)	2.54 (0.84)	2.32 (1.03)	1.86 (0.95)

^a^N/A: not applicable.

## Discussion

### Principal Findings

Previous intervention research in chronic pain has not comprehensively assessed improvement in physical function and has yielded modest improvements in self-reported emotional and physical function. To address this problem, we used evidence-based frameworks for intervention development and adaptation [21,22] and recent recommendations for assessment of physical function in chronic pain clinical trials [17-19] to iteratively develop *GetActive* *and GetActive-Fitbit*, 2 identical mind-body physical activity programs (8 sessions each) aimed at improving physical (self-reported, performance based, and objectively measured) and emotional function among patients with heterogeneous chronic pain by teaching them pain-specific and mind-body skills, and to gradually increase their activity in a manner noncontingent on pain levels [23]. In this study, we report on a feasibility RCT of the final iterations of these programs (10 sessions each) necessary before a future RCT to determine the efficacy of the 2 programs compared with a control and usefulness of the Fitbit in improving activity.

Consistent with our first hypothesis and the goals of both the ORBIT and NCCIH intervention development stage models, both programs met or exceeded the a-priori set feasibility benchmarks. Retention was considerably higher than other mind-body trials for chronic pain [68]. The establishment of these feasibility benchmarks is critical before efficacy testing to ensure the scientific rigor of the future efficacy trial, per the ORBIT and NCCIH stage models of intervention development. Leaping to efficacy testing before establishing such feasibility markers may have numerous, substantial, and negative unintended consequences, including having insufficient power to detect change, inadequate fit to the target population, and lack of identification or inclusion of those who are most likely to be most responsive to the intervention [69,70]. These results indicate that *GetActive* and *GetActive-Fitbit* are ideally poised for efficacy testing.

Consistent with our second hypothesis, we found that participation in both programs is associated with improvement in physical function. As this is not an efficacy trial, we limited analyses to effect sizes for within-group changes between pretest and posttest and refrained from between-group comparisons [61]. Both groups exhibited significant and large effect sizes for improvements in performance-based physical function (6-min walk test) and significant medium effect sizes in improvements in self-reported physical function. For objectively measured physical function assessed by ActiGraph, as a group, participants did not exhibit step count increases following the programs. However, individuals who were sedentary at baseline increased their step count over the MCID. Although any statements about efficacy are spurious with this small sample, the results suggest that sedentary individuals may be more likely to meaningfully increase step count compared with their nonsedentary counterparts. Indeed, only 2 individuals in the *GetActive* group and 1 in the *GetActive-Fitbit* group*,* who had step counts higher than 5000 at baseline, exhibited clinically meaningful improvements at posttest. It is also possible that the results were affected by weather variations between the 2 assessment periods, as we ran groups in early fall or winter, and posttreatment assessment was conducted during worse weather than baseline. Although 1 week is the recommended assessment time, a longer monitoring assessment may better capture inherent weather variations. In addition, a clear plan for maintaining activity during bad weather with identification of specific places to walk (eg, gym and mall) should be emphasized in the future efficacy trial. Participants in both groups exhibited medium effect sizes for improvements in emotional function, and medium-to-large effect sizes for improvements in pain intensity (above the MCID for changes in both pain at rest and during activity), pain catastrophizing, fear of pain due to movement, mindfulness, and adaptive coping. Importantly, participants rated their perceived improvement on our main outcomes as *much improved*.

### Limitations and Strengths

This study has several strengths. First, we used evidence-based frameworks and mixed methods to iteratively develop our mind-body program and refine our methodology. The study utilized strong scientific rigor and an RCT design that minimized the risks of selection bias and confounding. The emphasis on feasibility markers in preparation for efficacy is an additional strength of the study, which helps ensure the scientific rigor of the next step of the efficacy trial. Second, this study is the first RCT that follows recommendations from recent IMMPACT [18,19] and ICF [17] criteria to comprehensively assess physical function using objective, performance-based, and self-reported measures in chronic pain trials [71]. Finally, this study found improvements in physical function [71], pain coping [72,73], and pain intensity [11,73], which are similar to or larger than those found in other mind-body interventions, suggesting that our mind-body physical activity program shows strong potential for efficacy. Certain limitations should also be considered. First, our screening criteria for level of activity allowed many participants with relatively high baseline ActiGraph step counts to enroll (Table 3). These relatively active participants seem to have benefitted less in terms of ActiGraph measured step gains, although they did benefit from improvement in other aspects, including the 6-min walk test, self-reported physical function, and other intervention targets. Furthermore, although patients were instructed not to make changes in their activity during the baseline assessment, several shared in the group sessions that they did push themselves to be more active because they were excited about participation. Providing patients with a clearer rationale on why it is important to maintain their regular activity during the baseline assessment will be important to accurately capture objective activity. The last and largest cohort in our study experienced continuous rain during the postprogram ActiGraph assessment period, and all participants exhibited no or limited increase in step count, which may have skewed results. Fitbit step count data from *GetActive-Fitbit* further support this; Fitbit step counts have significantly increased above the MCID between week 1 and the final week of the program (+~1500 steps; *P=*.01; effect size of 0.52 SD units), but less so between week 1 and the week after program completion, when ActiGraphs were worn (~843 steps; *P=*.26; effect size of 0.23 SD units). In addition, 35% of the participants did not wear their ActiGraph at both baseline and posttest.

### Conclusions

The results of this study provide strong evidence that 2 novel mind-body and physical activity programs for patients with chronic pain are feasible, acceptable, credible, and yield high satisfaction. Furthermore, the programs show potential for improvement in physical function, pain and related coping, and other psychosocial variables. This study supports future testing of *GetActive* and *GetActive-Fitbit* as well as an educational control group in a fully powered 3-arm RCT to establish the efficacy of the programs in targeting physical and emotional function and determine whether reinforcing physical activity with a wearable digital monitoring device is beneficial in patients with chronic pain. This study provides a model for successfully following the IMMPACT criteria for a comprehensive assessment of physical function and following evidence-based models to maximize feasibility before formal efficacy testing.

## Appendix

Multimedia Appendix 1 GetActive and GetActive-Fitbit program development.

Multimedia Appendix 2 Participant flow.

Multimedia Appendix 3 Program content.

Multimedia Appendix 4 CONSORT-EHEALTH checklist (V 1.6.1).

## Abbreviations

CAMS-R: cognitive and affective mindfulness scale-revised
ICF: International Classification of Functioning, Disability and Health
IMMPACT: Initiative on Methods, Measurement, and Pain Assessment in Clinical Trials
MCID: minimum clinically important difference
MOCS: measures of current status
NCCIH: National Center for Complementary and Integrative Health
NRS: numerical rating scale
ORBIT: Obesity Related Behavioral Intervention Trials
PASIPD: self-reported physical activity scale for individuals with physical disabilities
PROMIS: Patient-Reported Outcomes Measurement Information System
RCT: randomized controlled trial
SMART: Specific, Measurable, Achievable, Relevant and Time-based
WHODAS: World Health Organization Disability Assessment Schedule
