# Supplementary figures and images for: A Mind-Body Physical Activity Program for Chronic Pain With or Without a Digital Monitoring Device: Proof-of-Concept Feasibility Randomized Controlled Trial
Source: JMIR Form Res. 2020 Jun 8;4(6):e18703. doi: 10.2196/18703 (PMC7308894; doi:10.2196/18703)

***
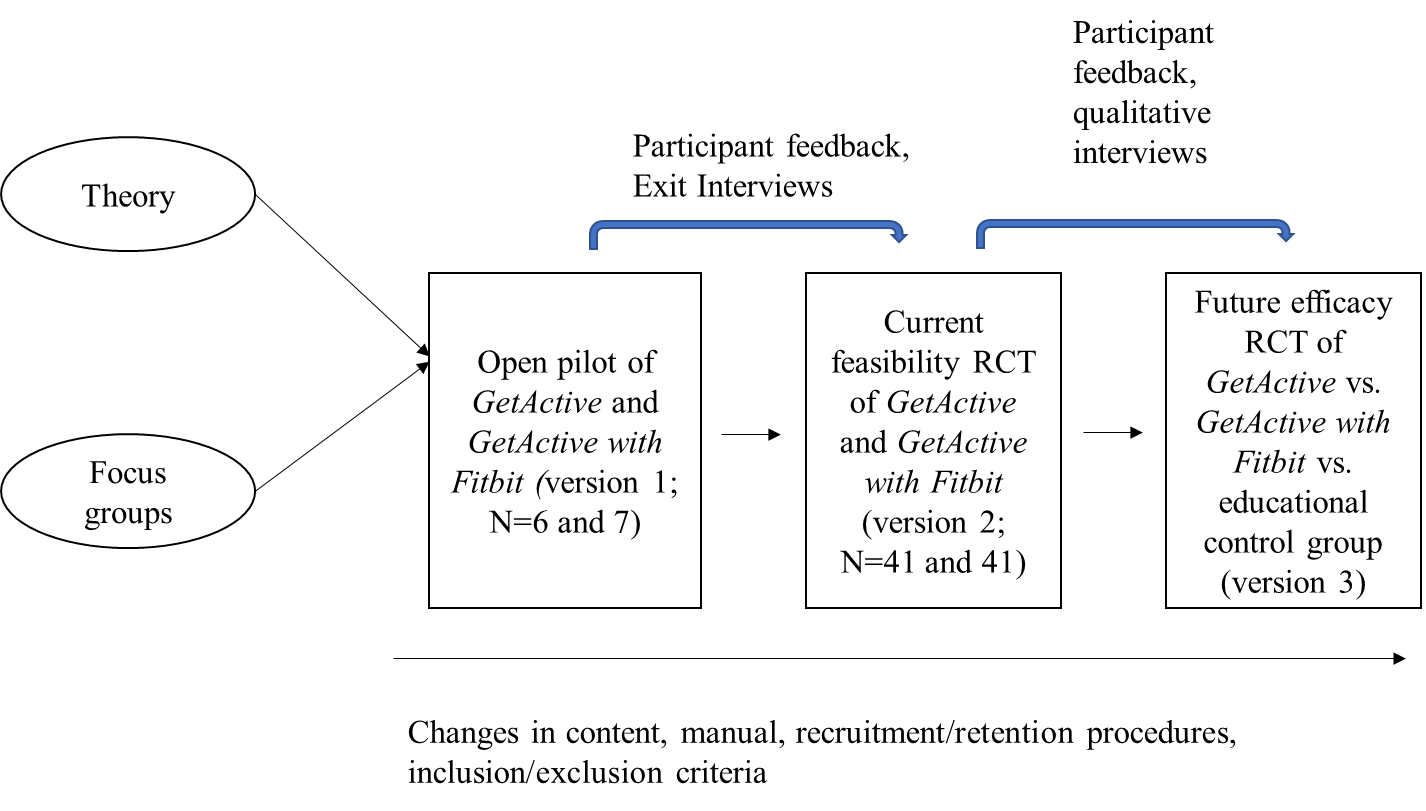
***

Supplement: Multimedia Appendix 1 [file formative_v4i6e18703_app1.docx]
