# Supplementary material for: A Mind-Body Physical Activity Program for Chronic Pain With or Without a Digital Monitoring Device: Proof-of-Concept Feasibility Randomized Controlled Trial
Source: JMIR Form Res. 2020 Jun 8;4(6):e18703. doi: 10.2196/18703 (PMC7308894; doi:10.2196/18703)
Supplement: Multimedia Appendix 2 [file formative_v4i6e18703_app2.docx]

Analyzed (n = 35)

Analyzed

Assessed for eligibility (n = 265)

Excluded (n = 183)

• 45 Active lifestyle

• 27 Current practice of mind-body techniques

• 13 Unable to walk without assistance

• 9 Does not have chronic pain diagnosis

• 12 No Bluetooth access

• 13 Current Fitbit use

• 4 not primarily English-speaking

• 2 Physician-imposed activity restriction

• 2 Recent medication dosage change

• 1 Active, uncontrolled psychosis

• 1 Deceased

• 63 Scheduling conflict

• 19 Lost to follow-up/contact concluded

• 42 Declined pre-screening

• 11 Declined post-screening

*Note: some participants are counted in multiple categories

Randomized to *GetActive-Fitbit* (n = 41)

• Received seven or more sessions (n = 34)

• Received less than seven sessions (n = 7)

Allocated to *GetActive* (n = 41)

• Received seven or more sessions (n = 29)

• Received less than seven sessions (n = 12)

Randomized (n = 82)

Allocation

Follow Up

Discontinued study (n = 6)

• 3 dropped prior to first session

• 3 dropped prior to completion

Analyzed (n = 37)

Discontinued study (n = 4)

• 1 dropped prior to first session

• 1 dropped prior to completion

• 1 discontinued due to hospitalization

• 1 admitted to rehabilitation and unable to complete post-test conditions
