# Supplementary material for: A Mind-Body Physical Activity Program for Chronic Pain With or Without a Digital Monitoring Device: Proof-of-Concept Feasibility Randomized Controlled Trial
Source: JMIR Form Res. 2020 Jun 8;4(6):e18703. doi: 10.2196/18703 (PMC7308894; doi:10.2196/18703)
Supplement: Multimedia Appendix 3 [file formative_v4i6e18703_app3.docx]

| Session | Topic | Skills |
| --- | --- | --- |
| Session 1 | Pain, Disability, and the Importance of Being Active | Pain Myths, The “Disability Spiral”, Deep breathing, Fitbit use (for *GetActive-Fitbit*), Building positivity with gratitude |
| Session 2 | Skill-Based Approach to Getting Active | Quota-based activity pacing, Choosing meaningful activities, SMART goals, activity barriers, Adherence to Fitbit (for *GetActive-Fitbit*) |
| Session 3 | Relaxation Skills to Manage the Pain Alarm | “True” or “false” pain alarm; Single pointed focus on the breath, Body scan, “MINIs” (brief meditation exercises) |
| Session 4 | Mindful Awareness and Pain | Mindful awareness, Pain meditation, Walking meditation for pain |
| Session 5 | Social Support and Chronic Pain | Identifying types of social support, Identifying social reactions to pain, The “pain cycle” |
| Session 6 | Mending the Chronic Pain Mind and Body | Identifying pleasant activities, Identifying negative automatic thoughts (NATs) |
| Session 7 | Creating an Adaptive Perspective | Guided imagery, Adaptive thinking, “Stop, breathe, reflect, choose” and links to chronic pain |
| Session 8 | Promoting Positivity | Loving kindness meditation, Optimistic Storytelling, Identifying relaxation signals, Getting back on track. |
| Session 9 | Healing States of Mind | Problem solving and Acceptance, Mindfulness of thoughts, Empathy, Contemplation and how they help with pain management and activity. |
| Session 10 | Humor, Empathy and Staying Resilient | Humor and laughter, Staying resilient for pain management, Overview of resiliency skills |
